# Minimally Invasive Surgical Resolution of Omental Infarction: A Case of Refractory Abdominal Pain

**DOI:** 10.7759/cureus.108445

**Published:** 2026-05-07

**Authors:** Amanda I Wosiack Menin, Militza Romagnoli Razmilic, Luana Wosiack Menin, Ramiro Caballero

**Affiliations:** 1 Surgery, El Carmen Dr. Luis Valentín Ferrada Hospital, Santiago, CHL; 2 General Surgery, El Carmen Dr. Luis Valentín Ferrada Hospital, Santiago, CHL; 3 General Medicine, Universidad de Santiago de Chile, Santiago, CHL

**Keywords:** acute surgical abdomen, diagnostic laparoscopy, minimally access surgery, omentum infarction, recurrent acute abdominal pain, refractory abdominal pain

## Abstract

Omental infarction is a rare cause of acute abdomen that frequently mimics more common surgical pathologies, often complicating the initial clinical assessment. We describe the case of a 35-year-old male patient with a history of hypertension and human immunodeficiency virus (HIV) infection who presented with severe, persistent abdominal pain in the right flank that was refractory to medical management. Notably, an initial computed tomography (CT) scan was negative, highlighting the diagnostic challenge of early presentation. Due to clinical persistence, a repeat CT scan performed 48 hours later was instrumental in confirming signs of omental infarction. The patient underwent an exploratory laparoscopy and resection of the necrotic omental segment, which confirmed the diagnosis of segmental omental infarction. Postoperatively, the patient showed favorable progress with rapid pain relief and was discharged two days after surgery. No further complications occurred during the follow-up period. This case underscores that while CT is essential for diagnosis, the clinical evolution dictates management, and an individualized laparoscopic approach allows for definitive confirmation and treatment, facilitating a quick recovery and potentially preventing long-term complications.

## Introduction

Omental infarction is a condition characterized by ischemia and necrosis of a portion of the greater omentum, a fat-laden peritoneal fold that protects and isolates intra-abdominal organs [1]. It can be caused by thrombosis or torsion of the omental vascular pedicle.

Omental infarction is a rare cause of acute abdomen, frequently diagnosed in adults with predisposing factors such as obesity, congenital venous anomalies, or increased intra-abdominal pressure [1,2]. Obesity has been identified as a risk factor due to the greater susceptibility of the omentum to vascular congestion [3].

The clinical picture usually presents with acute or subacute recurrent abdominal pain, the location of which depends on the affected omental segment [2]. However, it has been described in the literature that segmental omental infarction is mainly located in the right mid-abdominal area, which is explained by a congenital venous anomaly of the right omentum that predisposes to thrombus formation [1].

Approximately one-quarter of patients present with signs of peritoneal irritation, which can mimic surgical pathologies such as appendicitis, diverticulitis, epiploic appendagitis, or cholecystitis [2,4-6]. This can hinder the initial diagnosis and lead to unnecessary surgical interventions.

Computed tomography (CT) is the diagnostic tool of choice; it allows for the identification of specific characteristics of this condition, such as a slightly hyperattenuated fatty mass, oval or round in shape [7]. In cases of torsion, CT may show a "concentric stripes" pattern known as the "whirl sign," characteristic of this pathology [7]. Ultrasound can also be useful, showing a non-compressible hyperechoic mass located exactly below the site of maximum tenderness and directly under the abdominal wall [1].

The management of omental infarction varies according to the clinical presentation and radiological findings. In patients with a clear diagnosis based on imaging and without signs of complications (such as abscesses or peritonitis), conservative treatment is usually sufficient and includes analgesia, anti-inflammatories, and surveillance [3]. However, in cases of uncertain diagnosis, clinical deterioration, refractory pain, or unclear radiological findings, surgical intervention is recommended. Laparoscopy is the preferred approach, as it allows for confirmation of the diagnosis, resection of the affected segment, and minimization of associated morbidity [8]. Furthermore, surgical resection reduces the risk of long-term complications, such as the formation of intra-abdominal adhesions or abscesses [8].

The prognosis of omental infarction is generally favorable. Patients treated conservatively require radiological follow-up during the first months to rule out complications, while those undergoing surgery usually experience a faster recovery without the need for prolonged monitoring [7,8]. Despite being a known pathology, the rapid radiological evolution in less than 48 hours and the potential relationship with pro-thrombotic states, such as in patients with human immunodeficiency virus (HIV), highlight a clinical gap that this case report aims to address.

## Case presentation

We present the case of a 35-year-old male patient with a body mass index (BMI) of 28 kg/m² and a medical history of chronic arterial hypertension and HIV infection on long-term antiretroviral triple therapy. The patient consulted the emergency department for three consecutive days due to intense abdominal pain in the epigastric region, which had an acute onset and no other associated symptoms. Upon physical examination, he was in good general condition. Abdominal palpation revealed a soft, depressible, and painful abdomen in the right flank, but without signs of peritoneal irritation.

During his first emergency department consultation (day one), vital signs were stable: blood pressure (BP) 128/72 mmHg, heart rate (HR) 102 bpm, and temperature 36.7°C. Laboratory tests showed no inflammatory response, with a white blood cell (WBC) count of 5,900/µL (reference range: 4,500 - 11,000/µL), C-reactive protein (CRP) <0.1 mg/L (reference value: <5.0 mg/L), and a normal liver profile. A contrast-enhanced CT scan of the abdomen and pelvis was performed, which was entirely normal, specifically noting a normal appendix and no pathological findings. The patient was discharged with symptomatic management.

**Figure 1:**
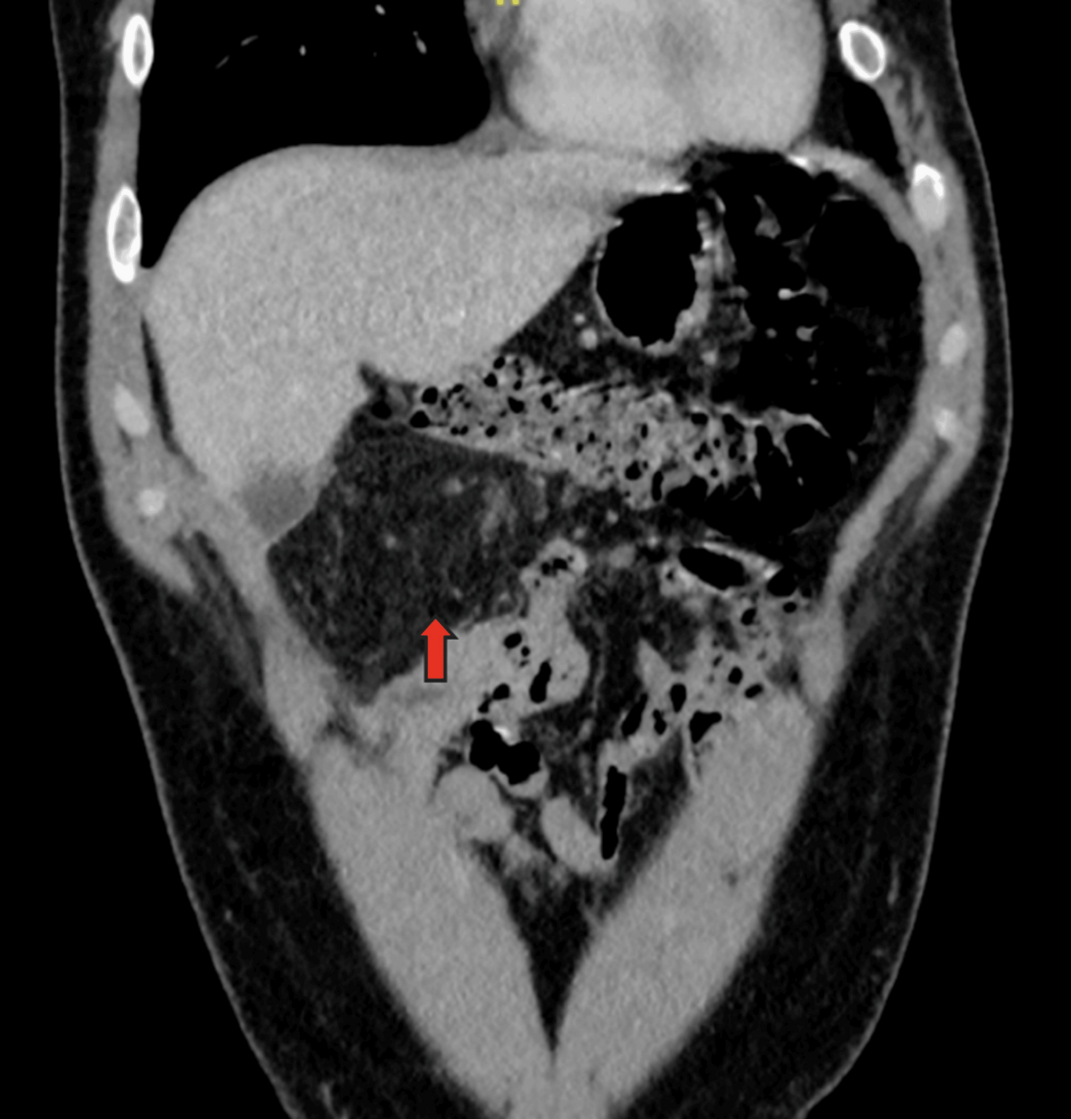
Contrast-enhanced CT of the abdomen and pelvis, coronal view A diffuse and ill-defined increase in the density of the omental adipose tissue in the right flank can be observed (arrow), suggestive of omental infarction.

Due to persistent pain, the patient returned on day two. New laboratory tests showed a slight inflammatory elevation (WBC 7,700/µL, CRP 8.8 mg/L). He was observed and discharged but returned on day three due to refractory symptoms. At this third visit, vital signs remained stable (BP 135/90 mmHg, HR 89 bpm, temperature 37.0°C), but inflammatory markers had increased (WBC 7,600/µL, CRP 38.7 mg/L). A non-inflammatory urinalysis was also noted. A second CT scan was requested, which reported "signs suggestive of omental infarction in the right flank" (Figure 1).

**Figure 2:**
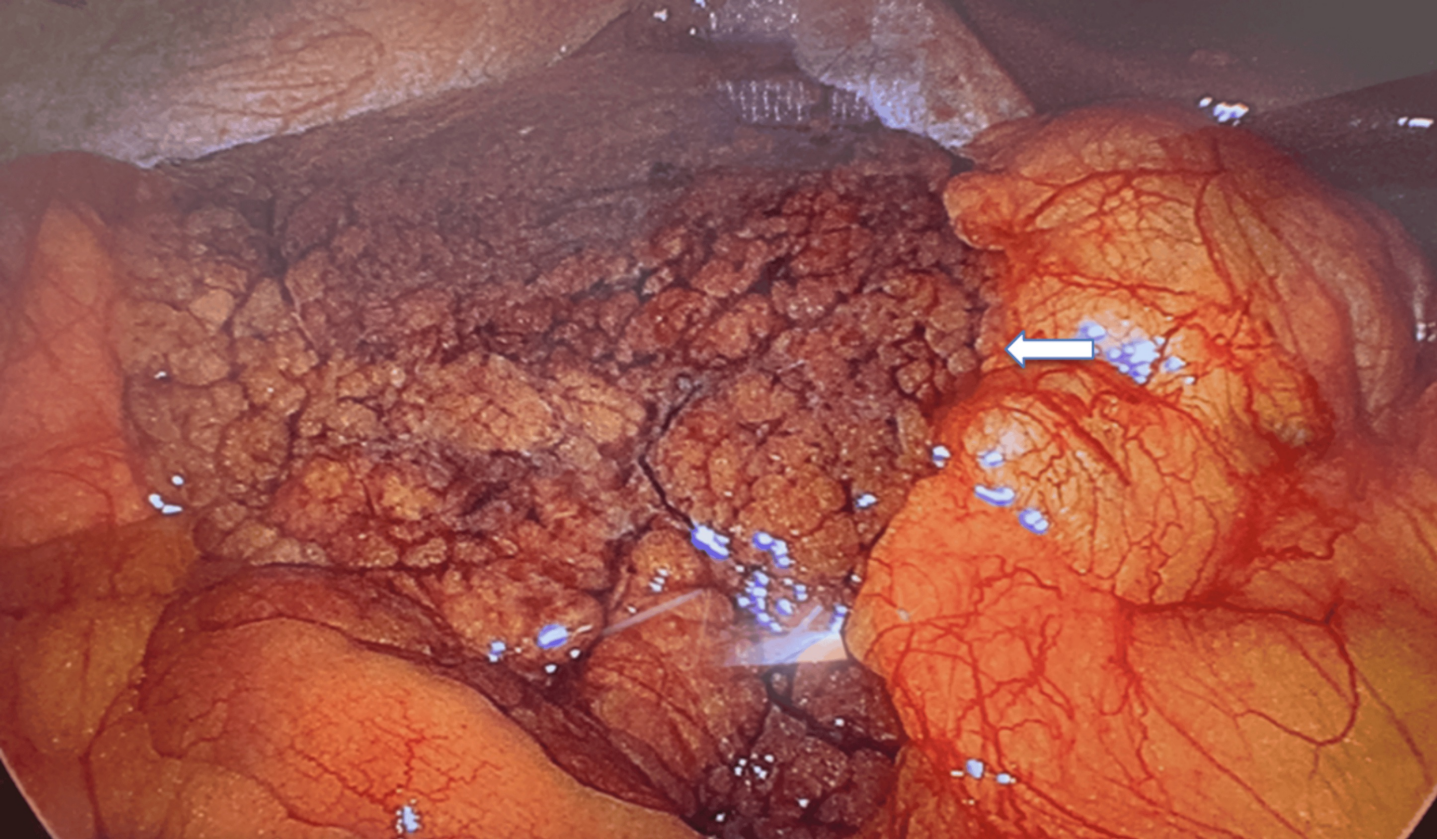
Intraoperative laparoscopic view The greater omentum is identified in the right flank, exhibiting a brownish-black coloration. The white arrow indicates the extensive area of infarcted and necrotic tissue.

Given that the omental infarction was of considerable size and the patient presented with refractory pain, an exploratory laparoscopy was performed. Using a French technique approach [9], a large portion of the greater omentum was found in the right flank. The tissue was necrotic, exhibiting a brownish-black coloration and high friability, associated with a mild hemoperitoneum (Figure 2).

**Figure 3:**
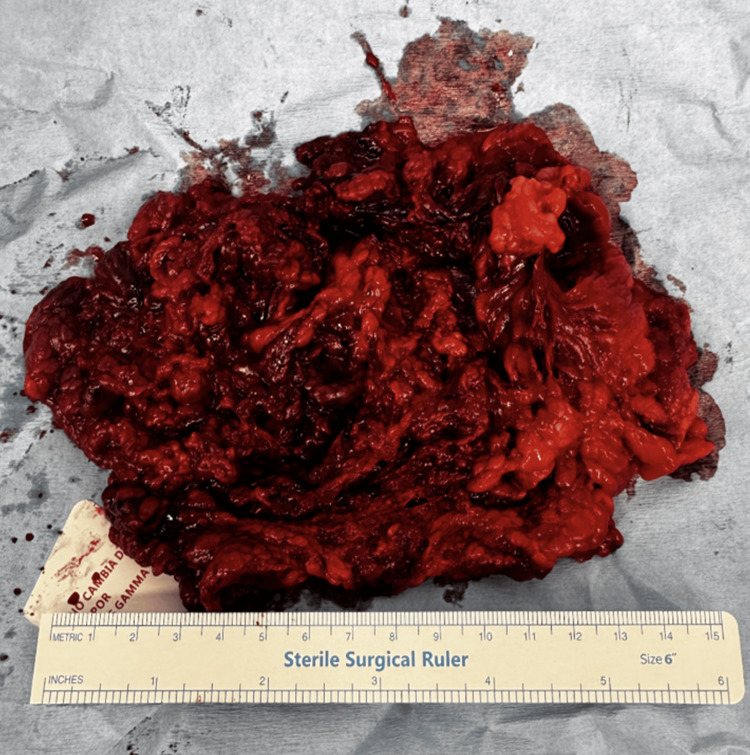
Macroscopic view of the resected segment of the greater omentum The surgical specimen demonstrated extensive hemorrhagic congestion and necrosis. It was subsequently sent for routine histopathological examination.

The infarcted portion was resected using advanced bipolar energy and extracted in an endobag. The surgical specimen was sent for routine histopathological examination (Figure 3), which later confirmed the diagnosis.

The patient's postoperative course was favorable, with a progressive reduction in pain. He was discharged two days after the procedure without complications. During the one-month outpatient follow-up, the patient showed a favorable clinical evolution with no additional complications. The final histopathology report confirmed the diagnosis of segmental omental infarction, showing extensive areas of fat necrosis and hemorrhagic congestion.

## Discussion

Omental infarction remains a rare and often elusive entity within the spectrum of acute abdominal pain. Its ability to mimic more common surgical conditions - such as acute appendicitis, cholecystitis, or diverticulitis - frequently leads to diagnostic challenges in the emergency department [4,5]. In our patient’s case, the clinical journey was particularly telling: the initial presentation with non-specific symptoms and a negative first CT scan could have easily led to a misdiagnosis. This underscores the importance of clinical persistence and the value of repeat imaging when symptoms do not follow the expected trajectory. The negative findings of the initial CT scan likely represent the ultra-early stage of ischemia rather than an interpretation error. This 'radiographic lag' is supported by the patient's initially undetectable CRP (<0.1 mg/L) and normal leukocyte count (5,900/µL) on day one, suggesting that significant tissue necrosis and perivascular edema had not yet developed to be tomographically visible. By day three, the rise in CRP to 38.7 mg/L mirrored the progression into a radiologically evident infarction.

Furthermore, it is important to consider the patient's medical history of HIV infection on long-term antiretroviral therapy (ART). While obesity is the most classically described risk factor, chronic HIV infection is known to be associated with endothelial dysfunction and a pro-thrombotic state. This underlying hypercoagulability, potentially exacerbated by long-term ART, could serve as an under-recognized predisposing factor for spontaneous vascular thrombosis in the omental pedicle, meriting further investigation in future case reports [10].

While CT is undeniably the gold standard for identifying the characteristic hyperattenuated fatty masses or the "whirl sign" of an infarcted omentum, the real clinical dilemma often lies in the management strategy [4,7]. Current literature frequently advocates for a conservative approach, centered on analgesia and close observation-especially in uncomplicated cases where the diagnosis is clear [3,7]. However, our experience suggests that a rigid conservative strategy has limitations. We propose concrete criteria to abandon medical management and transition to early laparoscopy: (1) pain remaining refractory after 48 hours; (2) a rapid upward trajectory of inflammatory markers (such as the CRP rise seen in this case); and (3) the presence of large infarcted segments or associated hemoperitoneum. Beyond definitive diagnostic confirmation, surgery allows for precise resection of necrotic tissue. The take-home message is that an initially negative CT scan must not override clinical persistence; dynamic clinical and laboratory evaluation should prompt a minimally invasive surgical resolution to provide immediate relief and potentially reduce long-term complications such as abscesses or adhesions [8].

Ultimately, the patient’s rapid recovery and discharge within 48 hours highlight the efficiency of minimally invasive surgery in this context. This case serves as a reminder that while conservative management is a valid starting point, an individualized surgical approach remains a crucial tool for the modern surgeon. It ensures that patients with significant symptomatic burdens or evolving clinical pictures receive definitive care that shortens hospital stays and optimizes long-term outcomes.

## Conclusions

Omental infarction is a diagnostic chameleon that requires a high index of suspicion, especially when common causes of acute abdomen have been ruled out. While radiology is a cornerstone of modern diagnosis, this case serves as a powerful reminder that clinical evolution and the patient's response to treatment must always guide our final decisions. Although conservative management remains the traditional first line of therapy, it should not be viewed as a rigid protocol. As demonstrated here, early laparoscopic intervention is a safe and highly effective alternative for patients with refractory pain or large infarcted segments. By choosing a minimally invasive surgical path, we can provide immediate symptomatic relief, significantly shorten hospital stays, and potentially reduce the risk of long-term sequelae of necrotic tissue, such as abscesses or complex adhesions. For the surgical team, the takeaway is clear: individualizing the approach is key to transforming a prolonged clinical struggle into a swift and successful recovery.

## References

[1] van Breda Vriesman AC, Lohle PN, Coerkamp EG, Puylaert JB (1999). Infarction of omentum and epiploic appendage: diagnosis, epidemiology and natural history. Eur Radiol.

[2] Liu G, Patel M (2021). A case of omental torsion and infarct diagnosed on computed tomography. J Med Imaging Radiat Oncol.

[3] Paroz A (2003). Idiopathic segmental infarction of the greater omentum: a rare cause of acute abdomen. J Gastrointest Surg.

[4] Danikas D, Theodorou S, Espinel J, Schneider C (2001). Laparoscopic treatment of two patients with omental infarction mimicking acute appendicitis. JSLS.

[5] van Breda Vriesman AC, Puylaert JB (2002). Epiploic appendagitis and omental infarction: pitfalls and look-alikes. Abdom Imaging.

[6] Pérez Saborido B, Jiménez Romero C, Marqués Medina E (2001). Idiopathic segmental infarction of the greater omentum as a cause of acute abdomen report of two cases and review of the literature. Hepatogastroenterology.

[7] Sánchez Fuentes PA, López López V, Febrero B, Ramírez P, Parrilla Paricio P (2015). Omental infarction: surgical or conservative management?. Cir Esp.

[8] Coppin T, Lipsky D (2006). Twisting and infarction of the entire greater omentum managed by laparoscopy: a report of two cases. Acta Chir Belg.

[9] Perissat J (1993). Laparoscopic cholecystectomy: the European experience. Am J Surg.

[10] Perkins MV, Joseph SB, Dittmer DP, Mackman N (2023). Cardiovascular disease and thrombosis in HIV infection. Arterioscler Thromb Vasc Biol.

